# A Deep Learning-Based Framework for Offline Robotic Weld Path Generation Using a Single Top-View RGB-D Image

**DOI:** 10.3390/s26154973

**Published:** 2026-08-05

**Authors:** Dahyeon Lee, Byungjin Ko, Taejoon Park, Jong-Wan Yoon, Homin Park

**Affiliations:** 1 Department of Applied Artificial Intelligence, Hanyang University, Ansan 15588, Republic of Korea; dahyeon11@hanyang.ac.kr; 2 Division of Smart Convergence Engineering, Hanyang University-ERICA, Ansan 15588, Republic of Korea; byungjinko@hanyang.ac.kr; 3 Department of Robotics Engineering, Hanyang University-ERICA, Ansan 15588, Republic of Korea; taejoon@hanyang.ac.kr; 4 Department of Intelligent Robotics, Hanyang University-ERICA, Ansan 15588, Republic of Korea; jongwanyoon@hanyang.ac.kr; 5 School of Science and Technology, Singapore University of Social Sciences, Singapore 599494, Singapore

**Keywords:** robot welding, weld seam extraction, RGB-D sensor, pipe welding, segmentation, robotic automation, computer vision

## Abstract

Pipe welding automation requires accurate weld seam extraction and reliable robotic weld path generation under complex geometric conditions. Existing vision-based approaches often rely on expensive laser sensing systems, multi-view sensing, or continuous seam tracking, resulting in increased hardware cost and system complexity. To address these limitations, this study proposes a deep learning-based offline robotic welding framework that generates a three-dimensional welding path from a single top-view Red–Green–Blue and Depth (RGB-D) image acquired prior to welding. The proposed framework integrates weld seam detection, semantic segmentation, morphology-based post-processing, RGB-D image alignment, coordinate transformation, and polynomial-based trajectory refinement into a unified pipeline for robotic weld path generation. A custom pipe welding dataset consisting of 1476 annotated images collected from representative industrial pipe materials with varying diameters was constructed to evaluate the proposed framework. The experimental results demonstrate that the proposed Region of Interest (ROI)-guided weld seam extraction pipeline improves the U-Net segmentation performance from 0.735 to 0.791 mean Intersection over Union (mIoU), while the detection model achieves a Recall of 0.988 and an mean Average Precision at an Intersection over Union threshold of 0.5 (${\mathrm{mAP}}_{50}$) of 0.995. Furthermore, polynomial-based trajectory refinement reduces the three-dimensional positional root mean square error (RMSE) to 0.333 mm, enabling continuous robotic welding over the entire visible weld seam without additional path modification. These results demonstrate that the proposed framework provides a practical and cost-effective solution for offline robotic weld seam extraction and weld path generation, while establishing a promising foundation for future extension toward online robotic welding through real-time weld seam tracking and adaptive trajectory correction.

## 1. Introduction

Welding is one of the most fundamental manufacturing processes and is extensively employed in industries such as aerospace [1], shipbuilding [2], construction [3], transportation [4], and automotive manufacturing [5]. Despite its widespread use, conventional manual welding still suffers from several limitations, including hazardous working environments, strong dependence on highly skilled operators, inconsistent welding quality, and reduced productivity caused by operator fatigue. These challenges have accelerated the adoption of robotic welding systems to improve manufacturing quality, operational safety, and production efficiency [6,7,8]. As industrial products become increasingly diverse and geometrically complex, robotic welding systems are required not only to automate the welding process but also to robustly identify weld seams and generate accurate welding paths under practical manufacturing conditions.

Conventional robotic welding systems generally generate welding paths using Teaching-and-Playback or Offline Programming (OLP) techniques based on predefined Computer-Aided Design (CAD) models. Although these approaches are effective in structured environments, they require considerable manual intervention during system setup and have limited adaptability to dimensional deviations caused by manufacturing tolerances or assembly errors [9]. To improve adaptability, numerous studies have investigated automatic weld seam extraction and welding path generation using image processing, laser sensing, sensor fusion, and more recently, deep learning techniques [8,10,11,12]. Image-processing-based methods provide low implementation cost and computational efficiency [13,14], whereas laser-based and sensor-fusion approaches achieve highly accurate three-dimensional weld seam localization [15,16,17]. More recently, deep learning techniques have significantly improved weld seam detection and segmentation performance in complex industrial environments [18,19,20]. However, these approaches often rely on expensive sensing hardware, multiple sensing viewpoints, or continuous seam tracking during welding, resulting in increased hardware complexity, computational cost, and limited practicality for industrial deployment. To overcome these limitations, this study aims to simplify the sensing configuration by utilizing only a single top-view Red–Green–Blue and Depth (RGB-D) image acquired before welding while maintaining reliable weld seam extraction and welding path generation performance.

Pipe welding presents additional challenges because workpieces exhibit curved surfaces, varying diameters, reflective metallic characteristics, and geometric deviations introduced during manufacturing and assembly [21]. These characteristics make robust weld seam extraction considerably more difficult than planar welding tasks, while sensing systems requiring continuous seam tracking further increase system complexity and reduce deployment efficiency in practical manufacturing environments. To address these challenges, the proposed framework performs weld seam extraction and welding path generation from a single pre-acquired RGB-D observation rather than relying on online seam tracking during welding. Furthermore, Region of Interest (ROI)-based weld seam detection suppresses irrelevant industrial background regions before segmentation, while morphology-based post-processing improves weld seam continuity and produces a refined weld centerline suitable for three-dimensional path generation.

To simultaneously address the aforementioned challenges associated with both existing weld seam extraction approaches and pipe welding environments, this study proposes an integrated framework for offline robotic weld path generation using only a single top-view RGB-D image acquired prior to welding. Rather than pursuing real-time weld seam tracking throughout the welding process, the proposed framework focuses on simplifying the sensing configuration while maintaining sufficient geometric accuracy for practical robotic welding applications. By combining ROI-based weld seam detection, semantic segmentation, morphology-based refinement, RGB-D image alignment, and polynomial-based trajectory correction into a unified pipeline, the proposed approach enables robust weld seam extraction and accurate three-dimensional welding path generation without requiring expensive sensing hardware, multiple sensing viewpoints, or continuous seam tracking.

**Figure 1:**
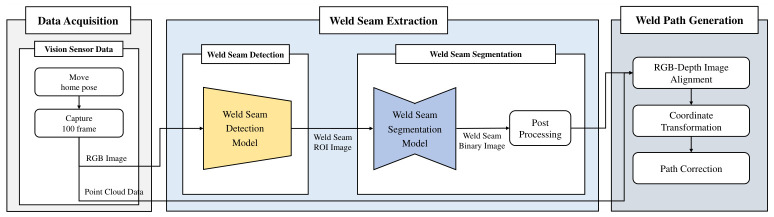
Proposed framework consisting of three subcomponents: data acquisition, weld seam extraction, and weld path generation.

To realize the proposed approach, this study develops the framework illustrated in Figure 1, which consists of three sequential subcomponents: data acquisition, weld seam extraction, and weld path generation. During data acquisition, a single RGB image and averaged depth information are simultaneously acquired using an RGB-D sensor before welding. The weld seam extraction stage integrates ROI-based weld seam detection, semantic segmentation, and morphology-based post-processing to accurately extract a continuous weld centerline. Finally, the extracted weld seam is transformed into three-dimensional coordinates through RGB-D image alignment and coordinate transformation, followed by polynomial-based path correction to generate an optimized welding trajectory. By integrating these components into a unified framework, the proposed method enables practical offline robotic welding path generation using only a single top-view RGB-D image.

The proposed framework was evaluated using a custom RGB-D dataset consisting of 1476 images acquired from five different pipe types. The ROI detection model achieved a Recall of 0.988 and an mean Average Precision at an Intersection over Union threshold of 0.5 (${\mathrm{mAP}}_{50}$) of 0.995, demonstrating reliable localization performance under various pipe geometries. By performing segmentation within the detected ROI, the segmentation accuracy improved from a Mean Intersection over Union (mIoU) of 0.735 to 0.784, while morphology-based post-processing further increased the mIoU to 0.791 by improving weld seam continuity and suppressing noisy regions. The resulting welding path achieved a root mean square error (RMSE) of 0.333 mm, demonstrating the feasibility of generating practical robotic welding paths from a simplified sensing configuration.

The main contributions of this study are summarized as follows:

1. We propose a deep learning-based offline robotic welding framework that generates a three-dimensional welding path using a single top-view RGB-D image, reducing reliance on expensive laser sensors and continuous seam tracking while enabling simplified sensing for robotic pipe welding automation.
2. We improve weld seam extraction performance by integrating an ROI-based detection module with morphology-based post-processing, improving the U-Net segmentation performance from an mIoU of 0.735 to 0.791 (approximately 7.6%) while reducing false detections caused by industrial background structures and enhancing weld seam continuity for robotic welding path generation.
3. We construct a custom RGB-D pipe welding dataset consisting of 1476 labeled images captured from five different pipe types to support weld seam extraction research for pipe welding automation.
4. The experimental results demonstrate that the proposed framework achieves reliable weld seam extraction and practical welding path generation performance with a three-dimensional positional RMSE of 0.333 mm.

## 2. Materials and Methods

The overall workflow of the proposed framework is illustrated in Figure 1. The framework consists of three sequential stages: (1) data acquisition, (2) weld seam extraction, and (3) weld path generation. The following subsections describe the implementation details of each stage.

### 2.1. Data Acquisition

The main objective of the data acquisition process is to obtain RGB images and Point Cloud Data (PCD) for accurate weld seam extraction. RGB images are used to analyze and identify visual characteristics of the weld seam, while depth information from the PCD is essential for determining its spatial location. The data are collected using a passive vision sensor mounted on the torch of the welding robot. The robot first moves to a predefined home pose that satisfies the camera’s minimum depth sensing range and ensures that the entire weld area is within the camera’s field of view.

To improve the reliability of the depth measurements, 100 consecutive depth frames are captured while the workpiece remains stationary, and the corresponding PCD are averaged to generate a single depth map. Unlike the depth information, only the final RGB frame is used because averaging RGB images can introduce motion blur caused by slight robot vibrations during image acquisition. The number of depth frames was determined through a preliminary evaluation using a calibration grid with 100 mm spacing between adjacent feature points. The average absolute coordinate errors were evaluated after transforming the measured camera coordinates into the robot coordinate system using different numbers of averaged depth frames (1, 10, 25, 50, and 100 frames). As summarized in Table 1, increasing the number of averaged frames consistently reduced the coordinate errors. The average errors along the x-, y-, and z-axes decreased from 10.745 mm, 6.982 mm, and 6.558 mm for a single frame to 0.012 mm, 0.031 mm, and 3.938 mm after averaging 100 frames, respectively. These results indicate that frame averaging effectively suppresses random depth fluctuations inherent to the RGB-D sensor.

The average z-axis error obtained with 100 frames averaging was reduced to 3.938 mm, which is already within the nominal depth accuracy (approximately 5 mm at a sensing distance of 1 m) specified for the RGB-D sensor used in this study. Therefore, additional averaging beyond 100 frames was considered unlikely to provide meaningful improvements in depth accuracy because the remaining error is primarily constrained by the intrinsic sensing accuracy of the camera rather than random measurement noise. Moreover, increasing the number of averaged frames would inevitably increase the data acquisition and processing time. Consequently, 100 frames averaging was adopted as an appropriate compromise between measurement reliability and computational efficiency for the proposed weld path generation framework.

### 2.2. Weld Seam Extraction

The weld seam extraction step consists of two subcomponents: weld seam detection and weld seam segmentation. The detection model identifies the ROI by generating a bounding box around the weld seam area from the input data. Using this bounding box, the input image is cropped and passed to the segmentation model. The segmentation model then produces a weld seam label image that provides a pixel-level classification of the weld seam.

#### 2.2.1. Weld Seam Detection

To minimize the time required for generating the weld seam path, it is essential to use a deep learning model with fast inference capability. One-stage detector architectures such as You Only Look Once (YOLO) provide significantly faster inference compared to two-stage detectors like Regions with CNN features (R-CNN). This advantage has made YOLO a popular choice for detection models in deep learning-based welding image recognition [8]. Among the various versions of YOLO, YOLOv8 was selected for its smaller model size, faster inference speed, and higher accuracy compared to YOLOv5.

To determine the most suitable YOLOv8 variant for the proposed framework, four models (YOLOv8-n, YOLOv8-s, YOLOv8-m, and YOLOv8-l) were experimentally evaluated in terms of detection accuracy and inference speed, as summarized in Table 2. Although all variants achieved identical Recall (1.000) and ${\mathrm{mAP}}_{50}$ (0.995), differences were observed in inference speed and localization accuracy measured by ${\mathrm{mAP}}_{50:95}$. YOLOv8-n achieved the fastest inference speed (8.7 ms/image), but its localization accuracy was lower than that of the larger models. In contrast, YOLOv8-m and YOLOv8-l increased the inference time to 19.7 ms/image and 27.8 ms/image, respectively, while providing no meaningful improvement in detection performance. Since the proposed framework performs semantic segmentation only within the detected ROI, accurate localization of the weld seam region is more critical than simply detecting its presence. Therefore, YOLOv8-s was selected because it achieved the highest ${\mathrm{mAP}}_{50:95}$ (0.779) while maintaining a relatively fast inference speed (13.3 ms/image), providing the best trade-off between localization accuracy and computational efficiency.

The YOLOv8-s model takes an RGB image acquired by the vision sensor, scaled to a size of 320 × 320 pixels, as its input. Its backbone network is based on the Cross-Stage Partial Network (CSPNet) structure, which enhances information flow, reduces computational cost, and improves feature learning. The neck module combines Feature Pyramid Network (FPN) and Path Aggregation Feature Pyramid Network (PAFPN) structures to effectively detect objects of various sizes by merging feature maps at different scales. This structure is further enhanced by a Path Aggregation Network (PANet), which strengthens the vertical flow of information and increases the utility of high-resolution feature maps.

The head module predicts the object class, bounding box, and objectness score. The model’s anchor-free design enables more flexible object detection without the need to pre-define the size and aspect ratio of anchor boxes. The data augmentation techniques are similar to those used in YOLOv5, although mosaic augmentation is excluded in the final epoch. The inference process produces bounding box coordinates for weld seam regions, using a confidence threshold of 0.7 that was determined empirically. These coordinates are then used to crop the corresponding regions from the original RGB image.

#### 2.2.2. Weld Seam Segmentation

For weld seam segmentation, this study adopts the U-Net model due to its strong performance and architectural suitability for the task. Comparative experiments with alternative models, including Fully Convolutional Network (FCN), U-Net, and DeepLabV3+, showed that U-Net consistently outperformed the others. This performance can be attributed to both the nature of the input data and the structural characteristics of the model. The cropped weld seam regions typically contain relatively simple linear or curved features. Therefore, a model with moderate depth and complexity such as U-Net is well suited to the task. In addition, the skip connections in the U-Net architecture support the effective fusion of spatial and semantic information. This helps improve segmentation accuracy and reduces the risk of overfitting.

The U-Net model used in this framework follows an end-to-end encoder–decoder architecture with three downsampling and upsampling stages. Its symmetrical design includes skip connections that link each encoder layer directly to its corresponding decoder layer. These connections enable the decoder to combine high-resolution features from the encoder with upsampled feature maps. This improves the reconstruction of spatial details in the final output. The encoder extracts multiscale features through successive convolutional layers, while the decoder progressively upsamples and refines these features to generate a segmentation mask that matches the resolution of the input. The input to the model is a weld seam ROI crop image, inferred by the weld seam detection model and resized to 320 × 320 pixels. The output is a binary mask in which weld seam pixels are labeled as 255 and non-weld areas as 0. This provides a pixel-level classification of the weld seam region.

#### 2.2.3. Image Post-Processing

**Figure 2:**
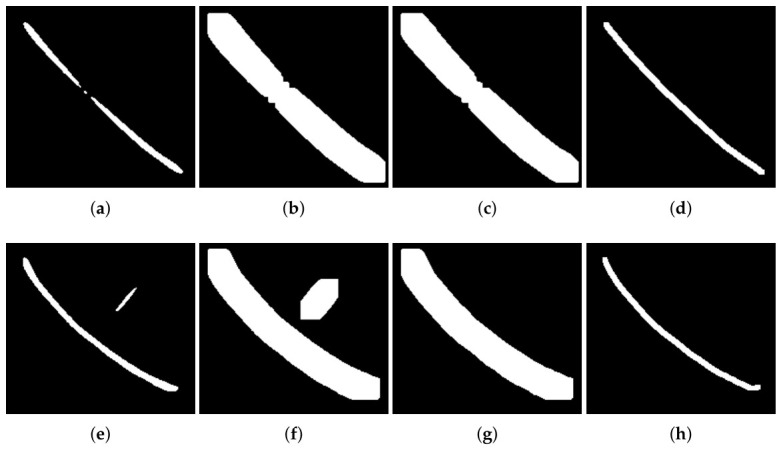
Image post-processing pipeline applied to weld seam mask images. (**a**,**e**) Segmentation outputs from the deep learning model; (**b**,**f**) after morphological dilation to enhance discontinuous weld regions; (**c**,**g**) after max contour filtering to remove noise; and (**d**,**h**) final results after thinning to extract centerlines for weld path generation.

The weld seam mask image generated by the deep learning segmentation model undergoes post-processing to enhance the reliability and accuracy of weld path extraction. While the model successfully predicted approximately 93% of the test dataset, which includes 148 images, there were occasional cases of broken or discontinuous weld seam representations, as well as false positives in non-weld areas. These problems are often caused by lighting conditions, material surface properties, and geometric complexities, especially around curved pipe regions. To address these challenges, a series of post-processing steps were applied, as illustrated in Figure 2. The procedure includes morphological dilation, max contour filtering, and thinning, applied sequentially to improve the structural integrity of the predicted mask and facilitate precise path generation.

The first step involves dilation, a morphological operation used to connect fragmented regions of the weld seam in the mask image. Discontinuities in the predicted mask may occur due to variations in surface reflectivity or shadowing effects, particularly at the peak points of pipe-shaped workpieces. The dilation operation enlarges the detected pixel regions to bridge these gaps and suppress small artifacts. A kernel size of 7 × 7 was used, and the operation was repeated three times. This configuration effectively reinforces the continuity of the weld seam while limiting the introduction of noise.

Following dilation, the max contour filtering technique is applied to eliminate extraneous regions incorrectly classified as weld seam. This method identifies the largest connected contour in the binary mask and retains it as the valid weld region, removing smaller regions often caused by scratches or background clutter. This filtering step is applied specifically to the cropped weld seam ROI image, rather than the full image, since the seam typically occupies the dominant area in the ROI. Applying the method to the ROI allows for effective noise removal without the risk of discarding valid weld seam regions, which could happen in full-sized images where background noise may be larger than the seam itself.

After contour filtering, thinning is performed to generate the center line of the weld seam, which serves as the basis for robotic path planning. Thinning reduces the weld seam to a single-pixel-wide representation, preserving the spatial trajectory of the seam. The resulting centerline coordinates are then mapped to the camera coordinate system to support accurate motion planning for the welding robot.

Together, these post-processing steps refine the initial segmentation output into a robust and executable weld path. Dilation reinforces discontinuities, max contour filtering eliminates irrelevant noise, and thinning extracts a streamlined center line for path planning. This processing pipeline enhances the precision and operational reliability of automated welding systems, enabling them to handle diverse workpiece geometries and variable imaging conditions in real-world environments.

### 2.3. Weld Path Generation

The weld path generation process involves creating robot paths based on the segmented weld seam areas. The process begins with image transformation techniques to align the coordinate systems of the skeletonized weld seam masks and the corresponding depth images. Once aligned, pixel coordinates corresponding to weld seams are extracted by identifying regions where the intensity value in the mask image equals 255. These coordinates are used to obtain the PCD in the camera coordinate system, which is subsequently transformed into the robot coordinate system. Using the resulting PCD associated with the weld seam, robot paths are generated in six degrees of freedom: x, y, z, roll, pitch, and yaw. The following outlines the detailed steps involved in the path generation process.

#### 2.3.1. RGB-D Image Alignment

**Figure 3:**
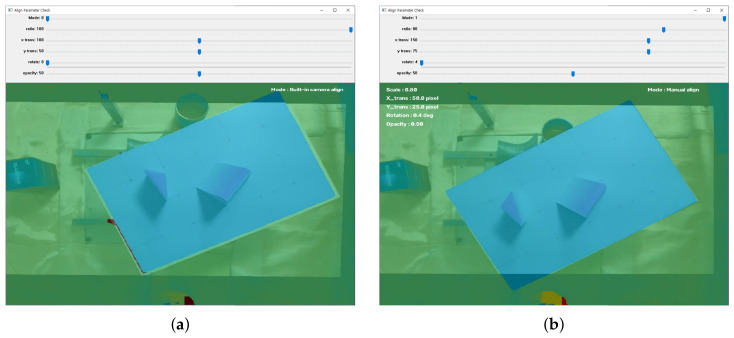
Comparison of RGB–D image alignment using the built-in camera alignment and the proposed manual alignment tool. (**a**) The result obtained using the built-in camera alignment provided by the RGB-D sensor; (**b**) the manually adjusted alignment achieved by tuning the scale, translation, rotation, and opacity parameters.

Accurate alignment of RGB and depth images is essential for reliable weld path generation, as it enables the association of depth values with the segmented weld seam pixels. Although the RGB-D sensor used in this study provides a built-in alignment function, visual inspection revealed significant misalignment, as shown in Figure 3a. To overcome this limitation, an in-house interactive RGB-to-depth registration tool was developed, as illustrated in Figure 3b. The tool directly acquires RGB and depth images from the connected RGB-D sensor and overlays them within a single display window. With the depth image fixed as the reference, the operator interactively registers the RGB image to the depth image by adjusting its scale, rotation, translations along the x- and y-axes, and overlay opacity while visually inspecting the registration results. Using this interface, the registration parameters providing the best correspondence between the RGB and depth images were manually determined.

**Figure 4:**
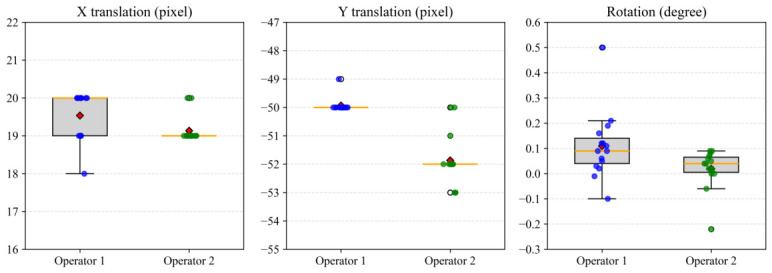
Distribution of the manually selected RGB-to-depth registration parameters obtained by two independent operators over 15 repeated calibration trials. The boxplots present the distributions of the X translation, Y translation, and rotation parameters.

To evaluate the repeatability of the proposed RGB-to-detph registration procedure, the initial values of the scale, rotation, and translation parameters were randomly initialized before each registration trial. Two independent operators then performed the registration procedure 15 times using the same RGB and depth image pair. To emulate the registration conditions expected for a different RGB–D sensor configuration, the relative transformation between the RGB and depth images was intentionally modified prior to the registration. Therefore, the registration parameters obtained in this experiment differ from those used in the main experiments. Table 3 and Figure 4 summarize the distributions of the selected scale, rotation, and translations along the x- and y-axes. The results show that both operators consistently selected highly consistent registration parameters with only minor variations. In particular, the scale parameter was consistently determined as 0.81 in all trials, while the translation and rotation parameters exhibited only small variations between repeated registrations. These results indicate that the proposed manual registration procedure provides good repeatability with low operator dependency. Based on the registration, the final parameters used in this study were a scale ratio of 0.8, a rotation angle of 0.4°, translation along the x-axes of 50 pixels, and translation along the y-axes of 25 pixels. These parameters were subsequently applied to align the skeletonized weld seam mask with the depth image, enabling accurate point cloud extraction for weld path generation.

Since the repeatability of the manual registration procedure was confirmed through the repeated registration experiments, the registration parameters only need to be determined once for a given hardware configuration. Once the relative positions of the RGB-D sensor, robot, and fixture (jig) are fixed, the registered parameters can be continuously reused without further adjustment, regardless of changes in the workpiece position or pipe type within the registered workspace. Therefore, the manual registration does not require repeated operator intervention during normal operation. Furthermore, the proposed RGB-to-depth registration tool was designed as a reusable registration utility rather than a sensor-specific implementation. When a different RGB-D sensor or production environment is used, only the RGB and depth image resolutions and the camera interface in the acquisition module need to be updated. The same interactive registration procedure can then be performed to determine a new set of registration parameters without modifying the remaining alignment workflow. Consequently, the proposed framework can be readily adapted to different RGB-D sensing systems while preserving the overall weld path generation pipeline.

#### 2.3.2. Coordinate Transformation

**Figure 5:**
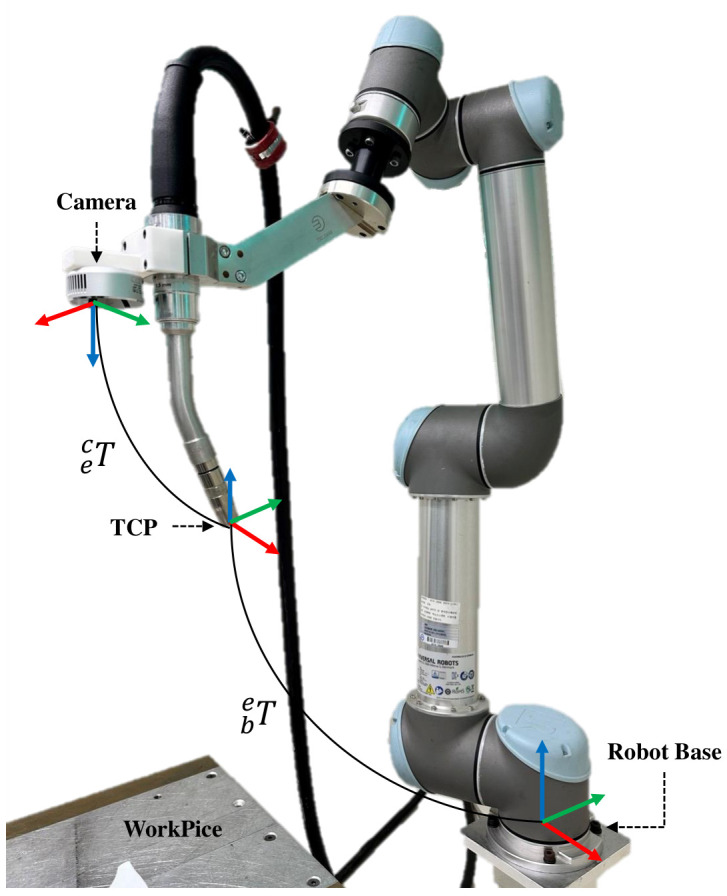
Coordinate frame transformations between the robot base, end-effector, and camera coordinate frames.

To convert the PCD from the vision sensor coordinate system to the robot base coordinate system, calibration between the vision sensor and the robot is required. In this study, eye-in-hand calibration is used, where the spatial relationship between the vision sensor and the robot’s Tool Center Point (TCP) is obtained through direct measurement. The transformation between the robot TCP and the robot base is determined using the built-in calibration functions provided by the Universal Robots (UR) UR5e robot. These functions incorporate the robot’s kinematic parameters, such as link lengths and joint angles, to establish a complete transformation pipeline. Using the acquired calibration data, a transformation matrix is constructed to convert coordinates from the vision sensor frame to the robot base frame, as illustrated in Figure 5. From the resulting point cloud in the robot coordinate system, only the points corresponding to the weld seam pixels identified are extracted. Finally, the robot path is generated by combining the 3D coordinates of these points with user-defined roll, pitch, and yaw values, yielding the final path representation in the format of $\left(x,y,z,r_{x},r_{y},r_{z}\right)$.

#### 2.3.3. Path Correction

**Figure 6:**
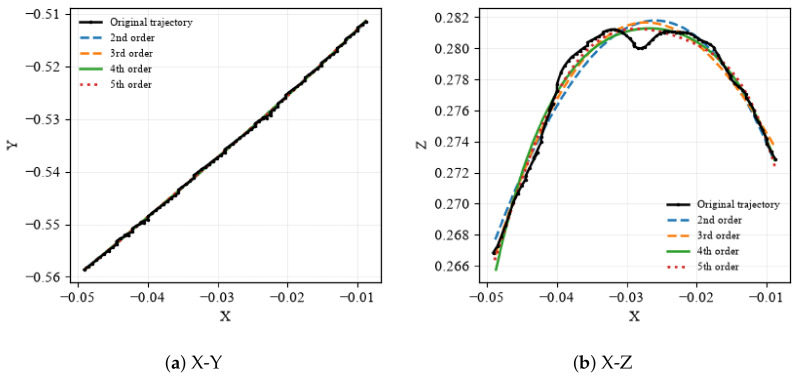
Comparison of polynomial fitting results with different polynomial orders for the generated robotic welding path. (**a**) Fitting results on the X–Y plane. (**b**) Fitting results on the X–Z plane. The black solid line denotes the original trajectory, and the colored curves represent the trajectories obtained using second-, third-, fourth-, and fifth-order polynomial fitting.

To improve the accuracy of the generated 3D robot path, a polynomial curve fitting method was applied. As the path is derived from the PCD obtained by the vision sensor, any inaccuracies in depth measurements can propagate to the final robot trajectory. As illustrated by the black dots and line in Figure 6, the raw path exhibits noticeable deviations in depth, resulting in an incomplete elliptical shape rather than the expected semi-circular geometry of the pipe weld seam. To correct these deviations, polynomial fitting was applied independently to the x-, y-, and z-coordinates using the NumPy polynomial fitting function, where the polynomial coefficients were estimated by the least-squares method.

### 2.4. Experimental Setup

#### 2.4.1. Hardware Configuration

Determining an appropriate polynomial order required additional experiments using second-, third-, fourth-, and fifth-order polynomial fitting. Figure 6 compares the resulting trajectories. The second-order polynomial produced an overly simplified trajectory that could not adequately represent the curvature of the pipe weld seam. In contrast, the fifth-order polynomial introduced unnecessary local oscillations caused by overfitting, despite closely following the sampled points. The third-order polynomial, as illustrated by the orange line, improved the overall trajectory but still exhibited slight deviations in regions with higher curvature. Among the evaluated models, the fourth-order polynomial generated the smoothest trajectory while accurately preserving the overall weld seam geometry, providing the best balance between geometric fidelity and trajectory smoothness. Therefore, a fourth-order polynomial was adopted for subsequent weld path generation.

The resulting correction significantly improved the continuity and accuracy of the generated welding path. After polynomial fitting, the local trajectory irregularities caused by depth fluctuations were effectively suppressed, producing a smooth and continuous trajectory suitable for robotic welding while preserving the overall weld seam geometry.

The hardware configuration was designed to ensure precision and efficiency in both model training and experimental validation. A UR5e collaborative robot [22] was employed to execute the welding paths, chosen for its flexibility and high positional accuracy under various experimental conditions. Welding operations were performed using the ABICOR BINZEL iROB Pulse 400 [23] with a custom jig securing the workpiece to maintain stability throughout the process. For vision-based data acquisition, an Intel RealSense L515 camera [24], captured RGB-D images, enabling accurate weld seam detection and path generation. The computing environment comprised two systems. Model training was conducted on an Ubuntu 18.04 workstation equipped with an Intel Xeon E5-2680 v4 CPU (2.40 GHz, 28 cores), a TITAN RTX GPU, and 125.6 GiB of memory. Physical experiments were executed on a Windows 10 system with an Intel Core i5-10600 CPU (3.30GHz) and a GeForce GTX 1080 Ti GPU. This configuration provided sufficient computational resources for deep learning development and satisfied the real-time demands of physical experimentation, ensuring consistent system performance throughout the study.

The pipe welding experiments were performed using a fixed-pipe configuration without rotary welding. Instead of rotating the workpiece, the welding torch attached to the UR5e collaborative robot followed the generated three-dimensional welding trajectory while the pipe was rigidly fixed by the jig. Two representative industrial pipe materials were used for the experiments: Stainless Steel 304 (SS304) pipes with a wall thickness of 3 mm and Steel Structure 400 (SS400) pipes with a wall thickness of 5 mm. All specimens employed a V-groove joint configuration. The detailed welding conditions used in the robotic welding experiments are summarized in Table 4. These welding parameters were kept constant throughout the experiments so that the evaluation focused on the accuracy and robustness of the proposed weld seam extraction and robotic weld path generation framework rather than variations in the welding process itself.

#### 2.4.2. Dataset

To evaluate the performance of weld seam detection and segmentation, a custom dataset was constructed using RGB-D images collected from five different pipes. Each pipe contributed approximately 300 images, except for one Stainless Steel 304 (SS304) pipe with an outer diameter (OD) of 60.6 mm, resulting in a total of 1476 images. As summarized in Table 5, the dataset includes three SS304 pipes and two Steel Structure 400 (SS400) pipes with different ODs. The pipe diameters were selected to represent commonly used industrial pipe sizes. In particular, a 114.3 mm (4-inch) pipe was selected as the reference diameter, as it serves as a global technical baseline (NPS 4/DN100) and is one of the most widely implemented sizes in international industrial piping systems due to its optimized fluid dynamics and extensive supply chain availability [25]. To incorporate geometric variations, both a smaller pipe (60.6 mm, 2-inch) and a larger pipe (165.2 mm, 6-inch) were additionally included to cover a comprehensive range of standard industrial scales.

For each pipe, the images were divided into training, validation, and test sets using an 8:1:1 ratio to ensure both training efficiency and generalization. The dataset was organized into three types. The first type contains original images paired with manually annotated ground-truth masks, created by manually labeling the weld seam regions. These images served as the primary training data for semantic segmentation. The second type includes images annotated with rectangular bounding boxes enclosing the weld seam regions for training the weld seam detection model. The third type consists of cropped ROI images extracted using the detected bounding boxes and was used to evaluate the effect of incorporating the detection module on segmentation performance.

**Figure 7:**
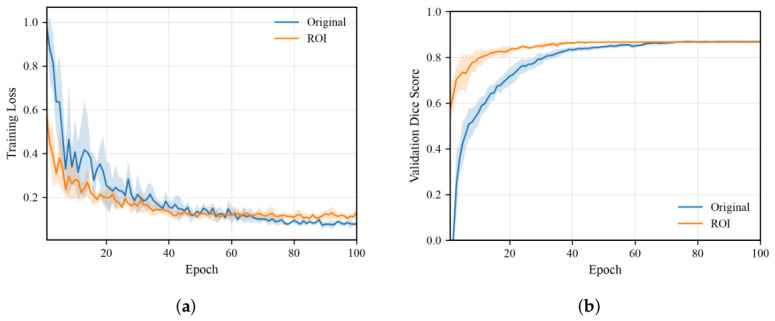
Training convergence of the U-Net segmentation model across five independent random seed experiments. (**a**) Training loss and (**b**) validation Dice score. Solid lines and shaded regions represent the mean and one standard deviation, respectively.

The performance reported in the following sections was obtained using models trained on the constructed dataset. Figure 7 presents the representative learning curves of the U-Net segmentation model for the original and ROI datasets. As shown in the Figure 7a, the training loss decreases steadily and converges to a stable value for both datasets. Similarly, Figure 7b shows that the validation Dice score converges without noticeable fluctuations during the later training stages. The consistent convergence of both the training loss and validation Dice score indicates stable optimization without evident overfitting, demonstrating that the constructed dataset is adequate for training the proposed segmentation model.

#### 2.4.3. Training Details

The training procedures were conducted on a NVIDIA Compute Unified Device Architecture (CUDA) -enabled system to ensure efficient computational performance. For weld seam detection, the YOLO-based model was trained for 80 epochs with a batch size of 16, using input images resized to 640 × 640 pixels. A pre-trained model was used as the baseline, and the initial learning rate was set to 0.01. The detection performance was evaluated using metrics such as precision, recall and mAP. For weld seam segmentation, a U-Net architecture was employed and trained for 100 epochs with a batch size of 4, using input images resized to 320 × 320 pixels. The optimization was performed using the Dice Binary Cross Entropy Loss function, and a cosine annealing scheduler was applied to dynamically adjust the learning rate during training. The segmentation model’s performance was assessed using the IoU, recall and 1-precision. Additionally, comparative experiments were conducted to analyze performance differences between models trained on full original images and those trained on cropped weld seam regions obtained from detection outputs.

## 3. Results

### 3.1. Performance Evaluation

#### 3.1.1. Performance of Weld Seam Detection Model

The proposed YOLOv8-s ROI detection model was evaluated using five different pipe types to verify its robustness in extracting weld seam regions from top-view RGB-D images. As summarized in Table 2, the proposed detector achieved a Precision of 0.997, a Recall of 1.000, an ${\mathrm{mAP}}_{50}$ of 0.995, and an ${\mathrm{mAP}}_{50:95}$ of 0.779, demonstrating highly accurate and reliable weld seam localization across various pipe geometries and material types.

**Figure 8:**
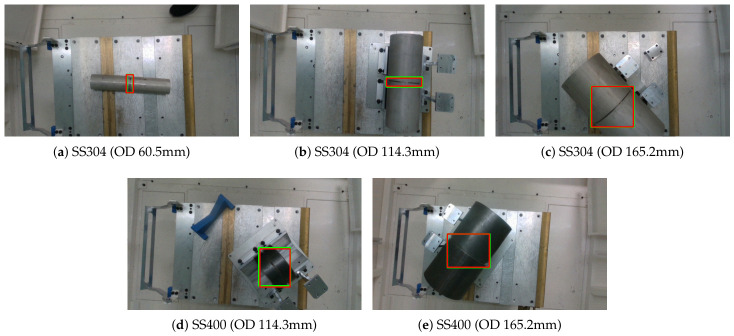
Representative successful weld seam detection results for five different pipe types. (**a**–**e**) Successful detection results for each pipe type. The red and green bounding boxes represent the predicted ROI and ground-truth annotation, respectively.

Representative detection results are shown in Figure 8. The predicted ROI (red bounding box) closely matches the ground-truth annotation (green bounding box) in all representative cases, indicating highly accurate localization of the weld seam region. Furthermore, the detector successfully distinguished the weld seam from surrounding linear structures, such as the fixture and other line-shaped components in the experimental testbed, which exhibit visual characteristics similar to the weld seam. These quantitative results and representative examples indicate that the proposed detector rarely missed weld seam regions or detected non-weld areas, while maintaining excellent agreement with the ground-truth annotations.

**Figure 9:**
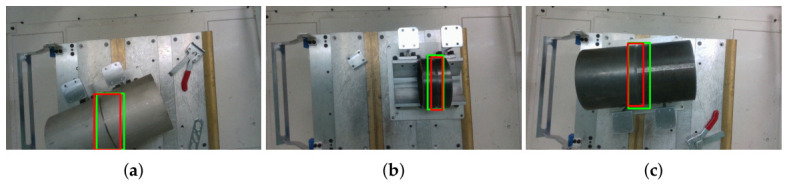
Representative ROI detection error cases showing minor localization differences between the predicted bounding boxes and the ground-truth bounding boxes. (**a**–**c**) Three representative examples of ROI detection results exhibiting slight localization differences. The red and green bounding boxes represent the predicted ROI and ground-truth annotation, respectively.

Although the proposed detector accurately localized the weld seam in the vast majority of the test images, several representative error cases exhibiting minor localization differences between the predicted bounding boxes and the ground-truth bounding boxes are shown in Figure 9. The slight differences between the predicted and ground-truth bounding boxes mainly originate from annotation variability. Since the objective of the ROI detector is to generate a region containing the entire weld seam rather than to estimate the exact object boundary, some manually annotated ground-truth bounding boxes were created with relatively loose margins, whereas others were tightly fitted around the weld seam. Consequently, a perfect overlap between the predicted bounding boxes and the manually annotated ground-truth bounding boxes is not always expected. Nevertheless, as illustrated in Figure 9a–c, all predicted ROIs successfully encompass the entire weld seam despite the minor localization differences. From the perspective of the proposed framework, these minor localization differences have a negligible effect on the subsequent segmentation stage because the predicted ROI still contains the complete weld seam. Therefore, the segmentation model can accurately segment the weld seam and generate the corresponding welding path without performance degradation.

Overall, these results demonstrate that the proposed ROI detector reliably generates bounding boxes that contain the entire weld seam despite minor localization differences with respect to the manually annotated ground-truth bounding boxes. Because the complete weld seam is preserved within the predicted ROI, these differences have a negligible effect on the subsequent weld seam segmentation process. Therefore, the detection performance achieved in this study is sufficient to support robust weld seam extraction and offline robotic weld path generation.

#### 3.1.2. Performance of Weld Seam Segmentation Model

**Figure 10:**
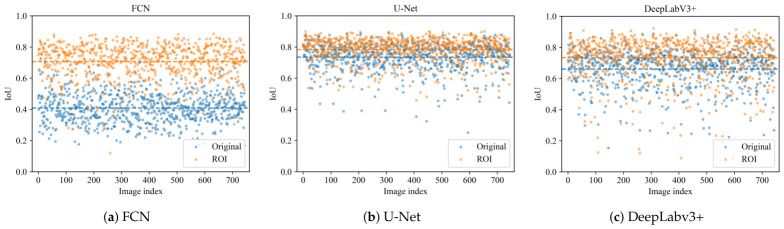
Comparison of IoU distributions between the original-image and ROI-based segmentation models.

To evaluate the robustness of the segmentation models, statistical analyses were performed using the IoU, Recall, and 1-Precision values obtained from five independent random seed experiments. Table 6 summarizes the mean and standard deviation of each metric, while Figure 10 compares the IoU distributions of the original-image and ROI-based models. Compared with the original-image models, all ROI-based models achieved higher mean IoU (mIoU) and Recall values while reducing 1-Precision, indicating that ROI extraction effectively suppresses false positive predictions caused by irrelevant background regions. Among all evaluated models, ROI-based U-Net achieved the best overall performance, with an mIoU of 0.784±0.075 and the highest Recall of 0.921±0.075. The scatter plots show that the IoU values of the ROI-based models are consistently shifted toward higher values compared with those of the original-image models, indicating that the performance improvement is observed throughout the test dataset.

The relatively poor performance of FCN can be attributed to its architectural limitations. FCN applies skip connections to only a subset of layers and performs single ×8 upsampling on a coarse 28×28 feature map, which often results in jagged segmentation boundaries. This design is particularly ineffective for tasks requiring fine-grained localization, such as weld seam segmentation. In contrast, DeepLabv3+, which employs a ResNet101 backbone, is designed to learn complex hierarchical features. Although this architecture is advantageous for datasets containing highly diverse object shapes and semantic information, it introduces unnecessary complexity for the relatively simple linear and curved weld seam patterns considered in this study. Consequently, DeepLabv3+ achieved better performance than FCN but did not outperform U-Net. Furthermore, all segmentation models converged more rapidly when trained using ROI images, demonstrating that eliminating irrelevant background information not only improves segmentation accuracy but also enhances training efficiency. Overall, U-Net provided the best balance between segmentation accuracy, prediction consistency, and learning efficiency, making it the most suitable model for weld seam segmentation in the proposed framework.

#### 3.1.3. Effect of Image Post-Processing

Morphology-based and image processing-based post-processing techniques were applied to enhance weld seam detection by refining the mask images predicted by the segmentation models. As shown in Table 7, applying post-processing led to improved mIoU scores across all models. Notably, the U-Net model achieved an mIoU increase from 0.784 to 0.791, highlighting its strength in capturing fine structural details and the added benefit of post-processing. Similarly, FCN and DeepLabv3+ showed performance improvements, with mIoU increasing from 0.707 to 0.723 and from 0.734 to 0.748, respectively.

**Figure 11:**
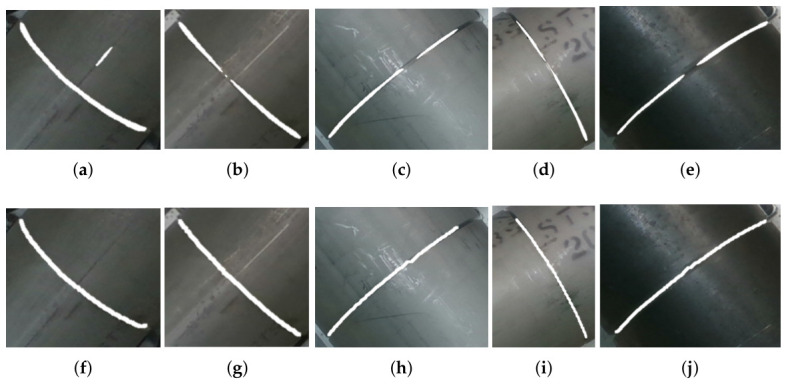
Comparison of weld seam segmentation results before and after image post-processing. (**a**–**e**) Raw segmentation outputs obtained from the detection module for five representative samples; (**f**–**j**) corresponding results after applying image post-processing.

Although weld seams typically appear darker than their surroundings, light reflections can cause certain segments to appear brighter, resulting in discontinuities in the predicted masks. Additionally, dark regions caused by scratches or pipe defects may be misidentified as weld seams. To address these challenges, morphological operations and the max contour method were applied. Morphological dilation reconnects broken weld seam segments, while the max contour method retains only the largest connected region in the mask, effectively removing small false positives. As shown in Figure 11, these techniques improved segmentation quality, especially in cases where the initial predictions were fragmented or noisy.

**Figure 12:**
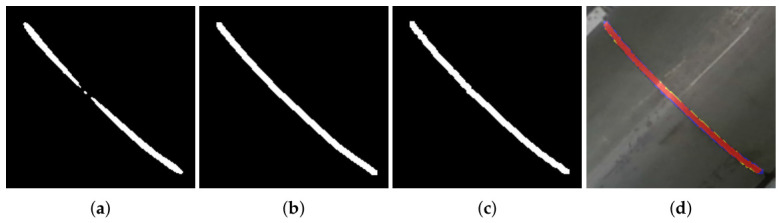

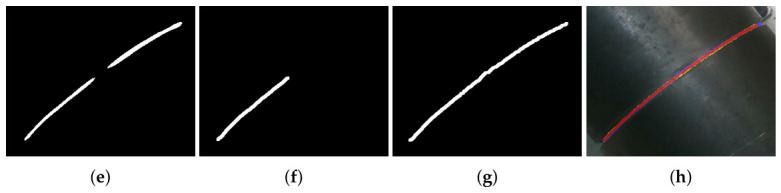
Comparison of post-processing results for weld seam mask images with and without consideration of discontinuous gaps. (**a**,**e**): Raw segmentation results predicted by the model; (**b**,**f**): post-processed results without considering the distance between broken weld segments; (**c**,**g**): post-processed results considering inter-segment distance by resizing and applying dilation; (**d**,**h**): final overlay images showing the corrected weld path on the original RGB image.

However, standard dilation operations have limitations. When the gap between disconnected segments is too large, dilation fails to bridge them, resulting in fragmented weld seams. Moreover, repeated dilation can blur fine details and alter seam thickness, reducing precision, as illustrated in Figure 12e,f. To overcome these problems, a distance-aware post-processing approach was introduced. Before applying dilation, the image was scaled down so that the distance between broken segments was reduced to 10 pixels or less, enabling a 7 × 7 kernel applied over three iterations to effectively bridge the gap. The resizing ratio was determined by calculating the minimum distance between bounding box coordinates of segmented contours. After resizing, dilation was performed, and the image was then restored to its original dimensions. As shown in Figure 12c,g, this distance-aware method successfully reconnected weld seams with wide discontinuities, further enhancing segmentation continuity and overall detection performance.

#### 3.1.4. Weld Path Error

**Figure 13:**
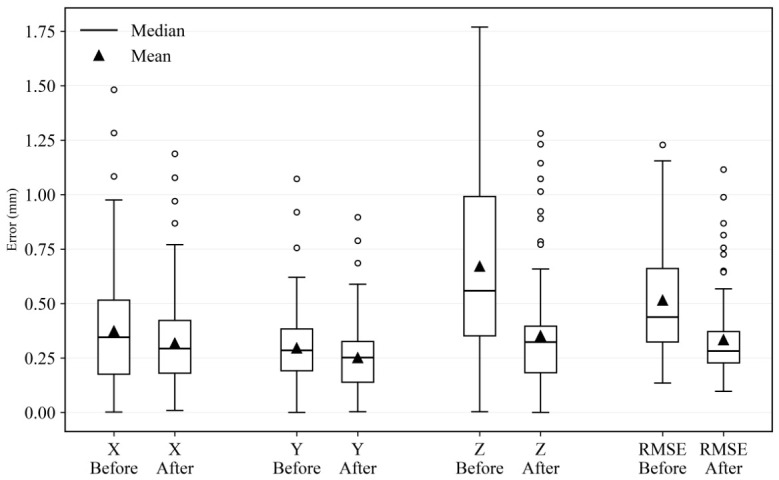
Box plots of the X-, Y-, and Z-axes welding path errors and the overall RMSE before and after polynomial curve fitting, evaluated over all 88 corresponding weld seam points. The horizontal line and triangle represent the median and mean, respectively.

To quantitatively evaluate the accuracy of the generated 3D welding path, the positional errors were computed by comparing the predicted robot trajectory with the ground-truth trajectory at all 88 corresponding evaluation points. The absolute errors along the x-, y-, and z-axes were analyzed individually. In addition, the three-dimensional positional RMSE was calculated for each evaluation point using the errors along all three coordinate axes. Table 8 presents the statistical distribution of the trajectory errors using all evaluation points. The evaluation reports the complete error distribution together with the mean, standard deviation, and minimum and maximum errors. After polynomial curve fitting, the mean error decreased from 0.374 mm to 0.318 mm along the x-axes, from 0.295 mm to 0.251 mm along the y-axes, and from 0.671 mm to 0.351 mm along the z-axes. The mean three-dimensional positional RMSE also decreased from 0.515 mm to 0.333 mm. Figure 13 shows that the interquartile range (IQR) becomes noticeably narrower after curve fitting, indicating that the proposed correction method not only improves the average accuracy but also reduces the variability of the generated welding path.

Among the three coordinate directions, the most significant improvement was observed along the z-axes, where the mean error was reduced by approximately 47.7%. Since the z-axes is directly associated with depth estimation from the RGB-D sensor, this result suggests that polynomial curve fitting effectively suppresses depth-related fluctuations and discontinuities in the reconstructed welding path. After correction, the z-axes error became comparable to the x-axes error, indicating that the proposed curve fitting effectively compensated for a large portion of the depth-related errors. Although the z-axes error became comparable to the x-axes error after correction, it remained slightly higher than those of the x- and y-axes. This residual difference is mainly attributed to the depth sensing characteristics of the RGB-D sensor when measuring narrow V-groove weld seams with highly reflective metallic surfaces.

Overall, the statistical analysis demonstrates that the proposed polynomial fitting method consistently improves both the accuracy and stability of the generated welding path. By evaluating all corresponding trajectory points instead of a limited subset, the revised results provide stronger evidence that the proposed framework effectively refines the generated welding trajectory while maintaining sub-millimeter average errors in the x- and y-directions and a substantially reduced depth error. These results support the practical applicability of the proposed framework for offline robotic pipe welding path generation.

**Figure 14:**
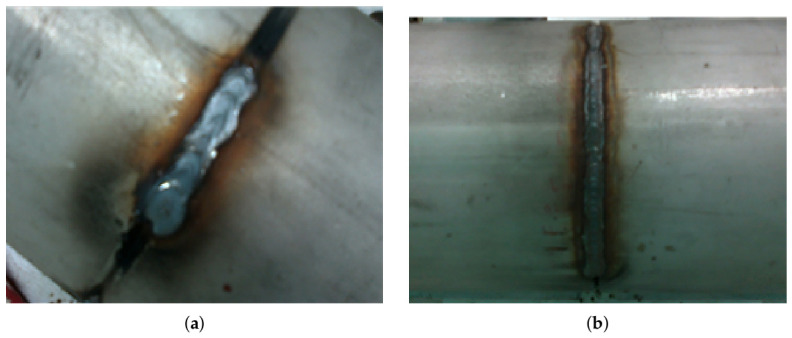
Comparison of robotic welding results before and after polynomial fitting: (**a**) before polynomial fitting and (**b**) after polynomial fitting.

In addition to improving the numerical accuracy of the generated trajectory, polynomial curve fitting also enhanced the practical usability of the welding path during robotic welding experiments. Before curve fitting, the generated trajectory frequently contained abrupt positional fluctuations near both ends of the pipe and around the central weld region due to segmentation noise and depth discontinuities. Consequently, the generated path often required manual trimming or trajectory regeneration before execution. Without these additional corrections, the welding robot was unable to continuously follow the entire weld seam visible in the top-view image, resulting in partial welding or incomplete weld coverage, as shown in Figure 14a. After applying polynomial curve fitting, these local trajectory fluctuations were effectively smoothed while preserving the overall weld seam geometry. As a result, the generated trajectory could be directly executed without additional manual post-processing, enabling continuous welding along the entire visible weld seam captured from the single top-view image. Figure 14b illustrates that the corrected trajectory produced a continuous weld bead covering the complete joint, demonstrating that the proposed fitting method improves not only trajectory accuracy but also the practical applicability of the proposed offline robotic welding framework.

## 4. Discussion

### 4.1. Comparison with Existing Approaches

Conventional weld seam extraction approaches commonly rely on laser scanners, structured-light sensors, or multi-view imaging systems to obtain accurate three-dimensional weld seam information. Although these approaches generally provide high precision, they often require expensive sensing hardware, complex calibration procedures, and large computational resources. In contrast, the proposed framework generates welding paths using only a single top-view RGB-D image while maintaining a weld path RMSE of 0.333 mm. This demonstrates that practical weld seam extraction can be achieved using a simpler and lower-cost sensing configuration.

In addition, previous studies on welding automation have mainly focused on flat workpieces or structured industrial environments. Pipe welding introduces additional challenges because curved surfaces, varying diameters, and reflections can significantly affect seam extraction performance. The proposed framework addresses these challenges by combining ROI-based detection and segmentation with morphology-based refinement techniques. The experimental results indicate that combining object detection and segmentation is effective for suppressing false-positive predictions in complex industrial scenes. This suggests that region-focused segmentation strategies can improve robustness in practical welding automation systems.

### 4.2. Industrial Applicability of the Proposed Framework

The proposed framework has practical potential for robotic pipe welding applications that require simplified sensing configurations and rapid welding path generation. Because the framework uses commercially available RGB-D sensors instead of high-cost laser scanning systems, the overall hardware cost can be reduced significantly. In addition, generating the welding path from a single image reduces sensing time and simplifies system integration compared with continuous seam tracking approaches. These characteristics make the framework particularly suitable for industrial environments where fast deployment, reduced setup complexity, and low-cost automation are important requirements. Furthermore, the proposed approach can potentially be integrated into collaborative robotic welding systems for semi-structured manufacturing environments.

Nevertheless, the industrial applicability of the proposed framework is currently limited to production environments that are sufficiently represented by the training dataset. Since the weld seam detection and segmentation models are data-driven, their performance may decrease when applied to previously unseen pipe geometries, joint preparations, groove configurations, or manufacturing defects that exhibit visual characteristics substantially different from those learned during training. Therefore, practical deployment in a new production environment is expected to require additional representative training data and model fine-tuning while maintaining the overall weld path generation pipeline.

The proposed framework was developed and validated using pipe configurations containing a single continuous weld seam. More complex pipe structures, such as T-, Y-, or cross-joints, may contain multiple weld seams within a single scene, requiring appropriate annotation strategies capable of representing multiple weld seams and corresponding training data for such configurations. In addition, the current post-processing algorithm was designed to extract a single weld seam by connecting fragmented regions through morphological dilation and retaining only the largest contour. Consequently, closely located weld seams may become unintentionally merged during dilation, while smaller but valid weld seams may be discarded during the largest-contour selection process. Extending the proposed framework to more complex pipe configurations may therefore require modifications to the weld seam extraction algorithm to reliably separate and preserve multiple weld seams for independent weld path generation.

The visual appearance of weld seams may also vary depending on the pipe diameter, wall thickness, and joint preparation. In particular, weld seams on small-diameter, thin-walled pipes without groove preparation may exhibit weaker visual contrast against the surrounding surface, making them more difficult to distinguish from other linear features. In such cases, positioning the RGB-D sensor closer to the weld seam to acquire higher-resolution images may improve the robustness of the proposed framework.

### 4.3. Future Work

Future research will focus on extending the proposed framework toward more practical robotic welding applications while preserving its simplified sensing configuration. Although the proposed framework demonstrated promising performance under the experimental conditions considered in this study, several challenges remain to be addressed.

One important research direction is to address the inherent occlusion limitation of single-view imaging. Rather than relying on a fixed multi-view sensing system, future work will investigate adaptive viewpoint planning strategies in which additional viewpoints are acquired only when the weld seam is partially occluded or cannot be reliably extracted from a single observation. Such an approach is expected to preserve the simplicity of the proposed framework while improving robustness for complex pipe geometries and self-occluded weld seams.

Another important direction is the development of automatic RGB–D image alignment techniques to replace the current manual parameter optimization procedure, thereby improving reproducibility and facilitating deployment with different RGB-D sensors.

Furthermore, although the present study focuses on offline weld path generation, the proposed deep learning-based weld seam detection and segmentation modules achieve sufficiently fast inference performance to support real-time perception. Therefore, the proposed framework provides a promising foundation for extending the current offline pipeline toward online robotic welding, where real-time weld seam tracking and adaptive trajectory correction can be incorporated during active welding processes. To further validate the proposed framework for practical industrial deployment, future work will also include weld quality assessment through appropriate metallurgical evaluations, such as macrosection analysis, thereby enabling comprehensive verification of both the generated weld path and the resulting weld quality.

## 5. Conclusions

This study proposed a deep learning-based framework for weld seam extraction and 3D welding path generation from a single top-view RGB-D image of a pipe workpiece. By using an RGB-D sensor instead of expensive laser-based sensing systems, the proposed framework provides a simpler and more cost-effective sensing configuration while maintaining the capability to generate a complete robotic welding path. The framework integrates ROI-based weld seam detection, semantic segmentation, morphology-based refinement, RGB–D alignment, and polynomial trajectory fitting into a unified weld path generation pipeline. To support the proposed framework, a custom RGB-D dataset consisting of 1476 annotated images collected from representative industrial pipe materials and diameters was constructed.

The experimental results demonstrated that incorporating ROI-based segmentation reduced background interference and improved the segmentation performance by approximately 7.6% in terms of mIoU, while morphology-based post-processing further increased the mIoU to 0.791. In addition, polynomial fitting improved the continuity of the generated welding trajectory, enabling continuous robotic welding over the entire visible weld seam without additional path modification. The generated welding path increased the three-dimensional positional RMSE to 0.333 mm. Overall, the experimental results demonstrate the feasibility of generating robotic welding paths using a single RGB-D sensor and a simplified vision-based framework for the representative pipe types and experimental conditions considered in this study. The proposed framework is designed to be adapted to different production environments through retraining with application-specific datasets while preserving the overall weld path generation framework.

## Figures and Tables

**Table 1:** Average absolute coordinate error according to the number of depth frames used for averaging.

Number of Frames	X Error (mm)	Y Error (mm)	Z Error (mm)
1	10.745	6.982	6.558
10	7.373	4.348	7.472
25	5.830	2.298	5.620
50	0.023	0.176	4.631
100	0.012	0.031	3.938

**Table 2:** Performance comparison of YOLOv8 model variants for weld seam ROI detection.

Model	Inference Time (ms/img)	Precision	Recall	${\mathrm{mAP}}_{50}$	${\mathrm{mAP}}_{50:95}$
YOLOv8-n	8.7	0.996	1.000	0.995	0.731
YOLOv8-s	13.3	0.997	1.000	0.995	0.779
YOLOv8-m	19.7	0.998	1.000	0.995	0.750
YOLOv8-l	27.8	0.998	1.000	0.995	0.729

**Table 3:** Repeatability evaluation of the manual RGB-to-depth registration parameters obtained by two operators over 15 repeated calibration trials.

Parameter	Operator 1	Operator 2
Scale ratio	0.810±0.000	0.810±0.000
X translation (pixel)	19.53 ± 0.64	19.13 ± 0.35
Y translation (pixel)	−49.93 ± 0.26	−51.87 ± 0.92
Rotation (°)	0.109 ± 0.134	0.019 ± 0.078

**Table 4:** Welding conditions used in the robotic welding experiments.

Parameter	Value
Welding process	Gas Metal Arc Welding (GMAW)
Welding power source	ABICOR BINZEL iROB Pulse 400
Welding mode	Synergic mode
Base material	SS304, SS400
Joint configuration	V-groove joint
Filler wire	KC-28 solid wire (EN ISO 14341-A G3Si1), (ϕ1.2 mm)
Shielding gas	82% Ar + 18% ${\mathrm{CO}}_{2}$
Welding current	80 A
Arc voltage	15.3–16.1 V (automatic in synergic mode)
Welding speed	8 mm/s
Wire offset	5 mm
Burn-back time	0.25 s
Pipe configuration	Fixed pipe (non-rotary welding)
Robot	UR5e collaborative robot

**Table 5:** Composition of the pipe weld dataset, including SS304 and SS400 pipes of multiple diameters. A total of 1180 training images, 148 validation images, and 148 test images were used in this study.

Pipe Type	Outer Diameter	Train	Valid	Test
SS304	60.5 mm	220	28	28
	114.3 mm	240	30	30
	165.2 mm	240	30	30
SS400	114.3 mm	240	30	30
	165.2 mm	240	30	30
**Total**		1180	148	148

**Table 6:** Performance comparison of segmentation models under different input conditions, including original images and ROI images.

Model	Input	IoU	Recall	1-Precision
FCN	Original	0.409±0.093	0.680±0.143	0.491±0.095
	ROI	0.707±0.109	0.870±0.111	0.179±0.087
U-Net	Original	0.735±0.087	0.889±0.086	0.110±0.074
	ROI	0.784±0.075	0.921±0.075	0.117±0.069
DeepLabv3+	Original	0.661±0.118	0.858±0.125	0.216±0.095
	ROI	0.734±0.130	0.901±0.141	0.131±0.083

**Table 7:** Comparison of segmentation performance after morphology-based post-processing over all test images. Values are reported as mean ± standard deviation.

Model	IoU	Recall	1-Precision
FCN (ROI)	0.707±0.109	0.870±0.111	0.179±0.087
FCN (ROI + Post)	0.723±0.008	0.773±0.008	0.143±0.009
U-Net (ROI)	0.784±0.075	0.921±0.075	0.117±0.069
U-Net (ROI + Post)	0.791±0.001	0.925±0.001	0.086±0.001
DeepLabv3+ (ROI)	0.734±0.130	0.901±0.141	0.131±0.083
DeepLabv3+ (ROI + Post)	0.748±0.004	0.865±0.004	0.110±0.003

**Table 8:** Descriptive statistics of the welding path errors before and after polynomial curve fitting.

Metric	Stage	Mean ± SD (mm)	Min (mm)	Max (mm)
X	Before fitting	0.374±0.270	0.002	1.482
X	After fitting	0.318±0.219	0.009	1.188
Y	Before fitting	0.295±0.176	0.001	1.072
Y	After fitting	0.251±0.161	0.003	0.896
Z	Before fitting	0.671±0.453	0.003	1.769
Z	After fitting	0.351±0.270	0.000	1.281
RMSE	Before fitting	0.515±0.251	0.135	1.229
RMSE	After fitting	0.333±0.183	0.097	1.115

## Data Availability

The dataset utilized in this study is available from the first author upon reasonable request for purposes of academic validation and non-commercial research.
